# Streptococcus thoraltensis Bacteremia: A Case of Pneumonia in a Postpartum Patient

**DOI:** 10.7759/cureus.5659

**Published:** 2019-09-14

**Authors:** Mohammed Wazir, Manmeet Grewal, Akriti G Jain, George Everett

**Affiliations:** 1 Internal Medicine, Florida Hospital, Orlando, USA; 2 Internal Medicine, Dayanand Medical College, Ludhiana, IND

**Keywords:** streptococcus spp, streptococcus, postpartum, gram positive cocci, non-pathogenic organism

## Abstract

*Streptococcus thoraltensis*, Genus *Streptococcus sensu stricto*, is a rare species of streptococci that has been very rarely reported to cause infection in humans. It is isolated from the sows and is found in the intestinal tract of pigs. We describe here a case of *S. thoraltensis* bacteremia in a postpartum patient with pneumonia.

## Introduction

Genus *Streptococcus sensu stricto* are Gram-positive cocci or short rods occurring in pairs or chains. They are nonmotile, nonsporing, catalase-negative, anerobic, and lactose fermenting [1]. *Streptococcus thoraltensis*, strain type S-69 (=LMG 13593) [2] is a recently identified strain of streptococci that has been isolated from the intestinal and genital tract of pigs and rabbits [3]. It is known to be nonpathogenic in humans.

## Case presentation

A 38-year-old female was admitted to the obstetric floor for an elective cesarean section (CS) complicated by pre-eclampsia, gestational diabetes, and history of two prior cesarean deliveries. She was discharged on postoperative day three after low transverse CS with bilateral salpingectomy.

**Figure 1:**
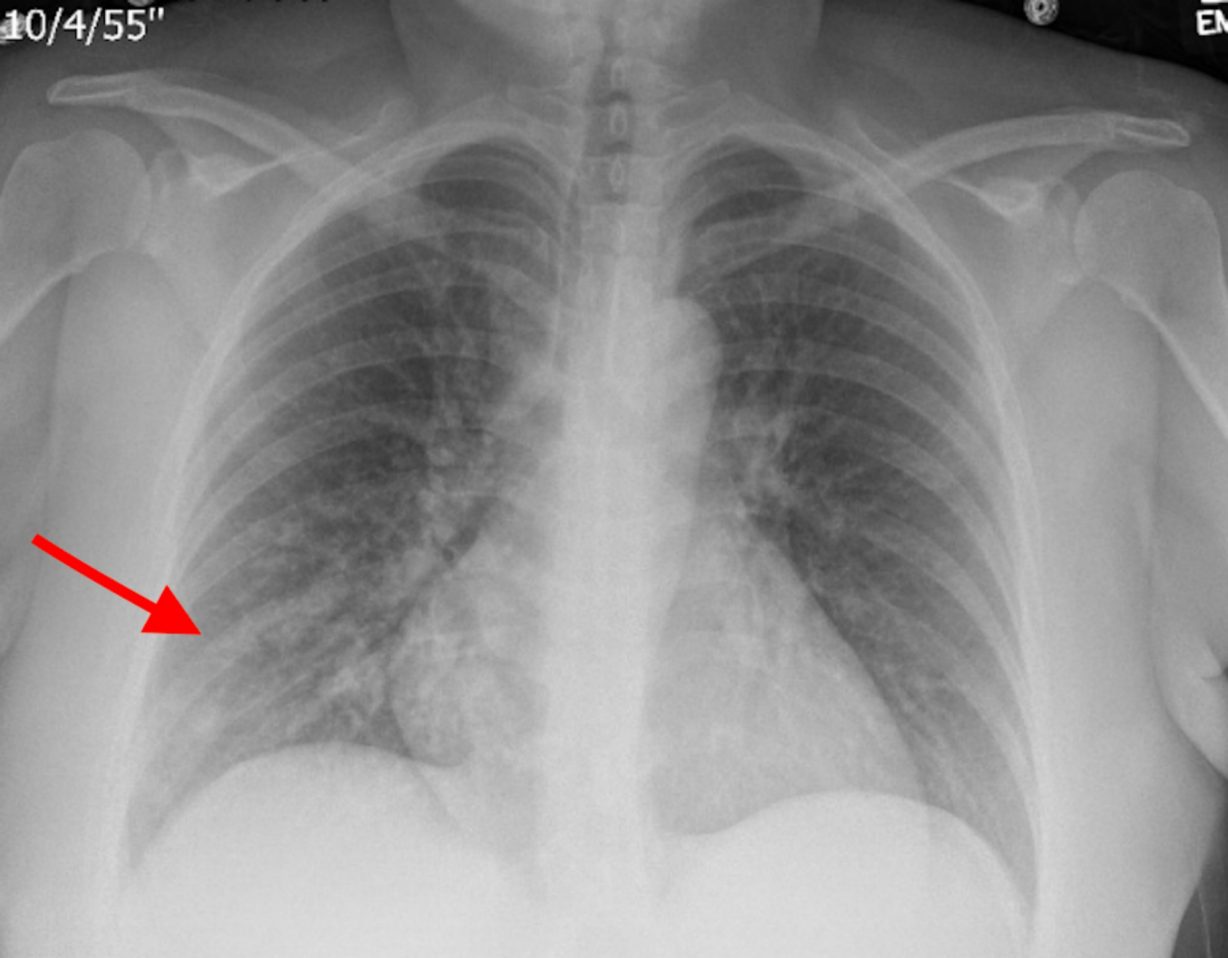
Chest X-ray showing patchy infiltrate in the right lower lung base (red arrow).

Five days later, the patient presented with three days of productive cough, headache, rhinorrhea, and subjective fever. The patient denied any history of abnormal vaginal discharge or uterine tenderness. The patient’s vital signs were significant for elevated blood pressure of 165/93 mmHg and bradycardia with heart rate in 50s bpm. She was not febrile; however, physical examination was notable for rhonchi heard in the right lung base. The cesarean incision was clean and dry with no signs of infection or dehiscence. Laboratory studies revealed normal white blood cell count, normal urinalysis, negative influenza screening, but elevated liver enzymes consistent with features of severe pre-eclampsia. Electrocardiogram (EKG) showed normal sinus rhythm. Chest X-ray (Figure 1) showed right lower lobe infiltrate suggestive of pneumonia. Blood cultures were taken and the patient was started on broad-spectrum intra-venous (IV) antibiotics in the form of cefepime and vancomycin. Blood culture showed Streptococcus species after 48 hours. Antibiotics were de-escalated to IV ceftriaxone. Transthoracic echocardiogram did not show any signs of infective endocarditis. On Day 4, the blood cultures were further identified as *S. thoraltensis* sensitive to penicillin, ceftriaxone, vancomycin, and fluoroquinolones. Sputum cultures as well as repeat blood cultures on Day 2 of hospitalization were negative.

The patient was discharged on IV ceftriaxone for 10 days. She recovered well with no complications or reoccurrence of symptoms on subsequent follow up.

## Discussion

The clinical significance of *S. thoraltensis* as a human pathogen remains uncertain. This unusual organism was first described by Devriese et al. in 1997 where it was isolated from the intestinal tracts of swine [2]. Few articles described the isolation of *S. thoraltensis* from the human oral and nasal cavities: a case of *S. thoraltensis* cultured from human subgingival plaque of a patient with severe perionditis [4]; and another study revealed *S. thoraltensis* to be among the predominant organisms colonizing the oral cavity and nasopharynx of 29 fuel workers [5]. Another case report revealed human infection by this organism in 2015 in a patient with chorioamnionitis where *S. thoraltensis* was isolated from both the maternal placenta and newborn tracheal aspirate cultures with history of paternal occupational exposure to pigs [6]. In 2018, Petridis et al. reported the first case of *S. thoraltensis* bacteremia in a patient with fever of unknown origin where the source of the infection remained undetermined. Unlike our case, their isolate was not susceptible in vitro to ceftriaxone but the patient responded well to treatment with ampicillin/sulbactam and gentamicin for 14 days [7].

According to a study conducted on patients during the first six weeks after delivery, 84% of re-admissions to the hospital were due to post-partum pneumonia [8]. Other cases of post-partum pneumonia have been attributed to pleuropneumonia-like organism present in genitourinary tract of man, methicillin-resistant *Staphylococcus aureus* necrotizing pneumonia arising from an infected episiotomy site, and aspiration pneumonia in the setting of eclampsia [9-10]. Lack of proper gold standard for the identification of streptococci [7] has posed a significant limitation for reporting of such strains and the subsequent challenge faced regarding the proper duration of treatment.

To our knowledge, this is only the second case report of *S. thoraltensis* bacteremia and the first reported case of pneumonia attributed to *S. thoraltensis*.

## Conclusions

Although *S. thoraltensis *has been predominantly described to cause disease in animals, its pathogenicity in humans has not yet been completely clarified and needs further exploration. We describe the second case in literature showing pathogenicity of this particular organism. Even though *S. thoraltensis* was previously considered nonpathogenic in humans, we propose that it can be pathogenic and cause disease pathology requiring treatment. Hence physicians should be cognizant of the pathogenic potential of this organism. 
